# Bioengineering Progress in Lung Assist Devices

**DOI:** 10.3390/bioengineering8070089

**Published:** 2021-06-28

**Authors:** Ahad Syed, Sarah Kerdi, Adnan Qamar

**Affiliations:** 1 Nanofabrication Core Lab, King Abdullah University of Science and Technology, Thuwal 23955-6900, Saudi Arabia; Ahad.Syed@kaust.edu.sa; 2 Biological and Environmental Science and Engineering Division, King Abdullah University of Science and Technology (KAUST), Thuwal 23955-6900, Saudi Arabia; Sarah.Kerdi@kaust.edu.sa

**Keywords:** artificial lung, respiratory failure, lung disease, membrane oxygenation, ventilation

## Abstract

Artificial lung technology is advancing at a startling rate raising hopes that it would better serve the needs of those requiring respiratory support. Whether to assist the healing of an injured lung, support patients to lung transplantation, or to entirely replace native lung function, safe and effective artificial lungs are sought. After 200 years of bioengineering progress, artificial lungs are closer than ever before to meet this demand which has risen exponentially due to the COVID-19 crisis. In this review, the critical advances in the historical development of artificial lungs are detailed. The current state of affairs regarding extracorporeal membrane oxygenation, intravascular lung assists, pump-less extracorporeal lung assists, total artificial lungs, and microfluidic oxygenators are outlined.

## 1. Introduction

The lungs are a vital part of the human respiratory system. It is the organ where the gaseous exchange occurs to maintain the basal functioning of the body. Breathing, which appears to be trivial, is quite a complex process. Air travel through the mouth and nose down to the trachea, which is divided into smaller passages called the bronchial tubes that go into each lung. The bronchial tubes branch out into smaller subdivisions throughout each side of the lung. The smallest branches are called bronchioles, and each bronchiole has an air sac, called alveoli. The alveoli have many capillary veins in their walls. Oxygen passes through the alveoli into the capillaries and the blood. It is carried to the heart and then pumped throughout the body to the tissues and organs. As oxygen goes into the bloodstream, carbon dioxide passes from the blood into the alveoli and then makes its journey out of the body, completing the gaseous exchange.

The lungs have a unique way of protecting themselves. Cilia, which look like a coating of tiny hairs, line the bronchial tubes. The cilia wave back and forth, spreading mucus into the throat so that the body can dispel it. Mucus cleans out the lungs and rids them of dust, germs, and any other unwanted items that may end up in the lungs. The lungs can have a wide range of problems stemming from genetics, bad habits, an unhealthy diet, and viruses. Diseases and conditions of the respiratory system fall into two categories: infections, such as influenza, bacterial pneumonia, and enterovirus respiratory virus, and chronic diseases, such as asthma and Chronic Obstructive Pulmonary Disease (COPD). COPD, sometimes called chronic bronchitis or emphysema, is an established and progressive disease where the airflow in and out of the lungs decreases, making it harder to breathe. Over time, the airways in the lungs become inflamed and thicken, making it harder to eliminate carbon dioxide.

**Figure 1:**
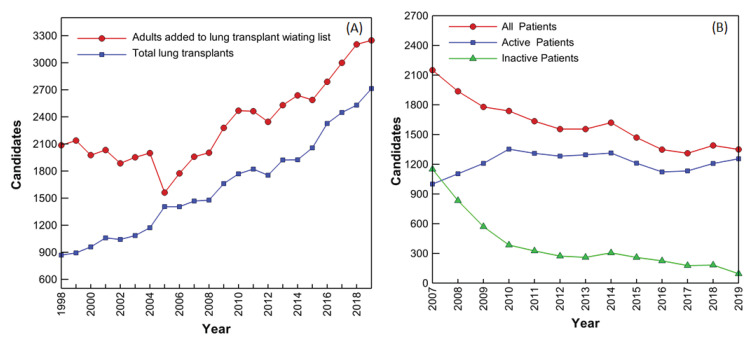
(**A**) Evolution of adult patient number added to the lung transplant waiting list and of total lung transplants since 1998 [1,2]. (**B**) Evolution of adult candidate number waiting for lung transplant [1].

Respiratory failure, the common terminus of lung diseases, is a leading cause of death in developed countries and is currently the third leading cause of death in the United States. Among respiratory diseases, COPD tops the chart with more than 15 million people suffering from it in the United States alone. The steady increase of COPD cases and the associated mortality is creating a grim picture around the world. The only long-term treatment for patients with end-stage lung disease is pulmonary transplantation, but transplant demands well exceeds donation rates (Figure 1A) [1]. Waiting list additions have been increasing steadily, after an initial decline immediately following implementation of the Lung Allocation Score (LAS) in 2005. The LAS is an attempt to identify the best candidates for transplant by estimating risk of death without transplant and post-transplant. In 2019, more than 3200 new candidates were added to the waiting list; this was the largest number of lung transplant candidates added to the waiting list in a single year since at least 1998 (Figure 1A) [1,2]. Year-end active wait-list counts have also been steadily increasing, (except for year 2016), indicating that donation and transplant rates have not been able to keep pace with the influx of new lung transplant candidates (Figure 1B).

These numbers are not helped by the infections caused by COVID-19 coronavirus which affects the alveoli causing pneumonia and in extreme cases Acute Respiratory Distress Syndrome (ARDS). We have already witnessed over 3 million mortalities associated with severe ARDS conditions created by Corona virus infections. The first successful lung transplant on a COVID infected patient has been performed in the United States and experts predict as younger patients get seriously infected, lung transplant would be the best way ahead for a long and fruitful life. As a result, a technology capable of supporting the function of a human lung while keeping them ambulatory is highly sought after. This technology, the smart artificial lung or smart ventilation system, may be on track to meet such demands.

The various devices which are today termed “artificial lungs” have a rich history of bioengineering innovation. The 19th-century realization that blood could be artificially oxygenated quickly gave way to a number of devices for perfusing single organs. These early oxygenators laid the groundwork for the heart-lung machines of the 1950s that allowed the first cardiopulmonary bypass surgeries. Advances in gas-permeable biomaterials allowed oxygenators to facilitate long-term perfusion in the 1970s, and by the second millennium extracorporeal life support technologies were quickly improving.

Today, extracorporeal membrane oxygenation (ECMO) is finding considerable success in bridging patients to lung transplantation. Other modern technologies include intravascular lung assists, pumpless extracorporeal lung assists, total artificial lungs, microfluidic oxygenators and Artificially Intelligent (AI) ventilation systems. These technologies have met varied success, but all are presently challenged to efficiently oxygenate blood without inducing thrombosis. After a review of the history, an update on the progress and future directions of each of these artificial lung techniques will be investigated.

## 2. Historical Progress

### 2.1. Prehistory of Clinical Oxygenators

In 1812, César Julien-Jean Le Gallois wrote “If one could substitute for the heart a kind of injection of arterial blood, either naturally or artificially made, one would succeed in maintaining alive indefinitely any part of the body whatsoever” [3]. Le Gallois [4] had attempted to supply blood to decapitated rabbit heads, but was thwarted by coagulation. Nevertheless, his stunning hypothesis remarkably achieved fruition in less than three decades. In 1849, Lobell [5] performed a successful isolated-organ perfusion by injecting defibrinated arterial blood into a kidney. In the 1850s, Brown-Séquard successfully reactivated the muscles of recently-guillotined criminals using blood transfusions from himself [6].

While transfusion medicine thereafter developed in its own right, Brown-Séquard first realized the potential for blood to be artificially oxygenated. Subjected to a vigorous beating process, he found that dark blood commingled with air turned red [6]. The medical implications of artificial oxygenation were thus recognized, and so began the search for a mechanical device that functioned as a lung. In 1865, Ludwig [7] attempted to oxygenate blood by shaking it within a balloon, but his setup lacked the efficiency needed to maintain continuous perfusion. In 1882, von Schröder [8] invented a device by which air bubbles forced blood from a venous reservoir into an arterial reservoir, and in doing so induced oxygenation. Scientists from the same lab, Frey and Gruber [9], in 1885 developed an apparatus in which a thin layer of blood was rotated within an inclined cylindrical drum. These designs, and the many variants that followed [10], oxygenated blood by exposing it directly to open air, and so were termed direct-contact oxygenators. Due to the blood trauma they induced, direct-contact oxygenators were not generally successful at perfusion until the 1916 discovery of herapin, an anticoagulant [11]. Through anticoagulants, however, the designs of Schröder’s bubble oxygenator and Frey and Gruber’s film oxygenator laid the groundwork for extracorporeal oxygenators used until the late 1970s.

**Figure 2:**
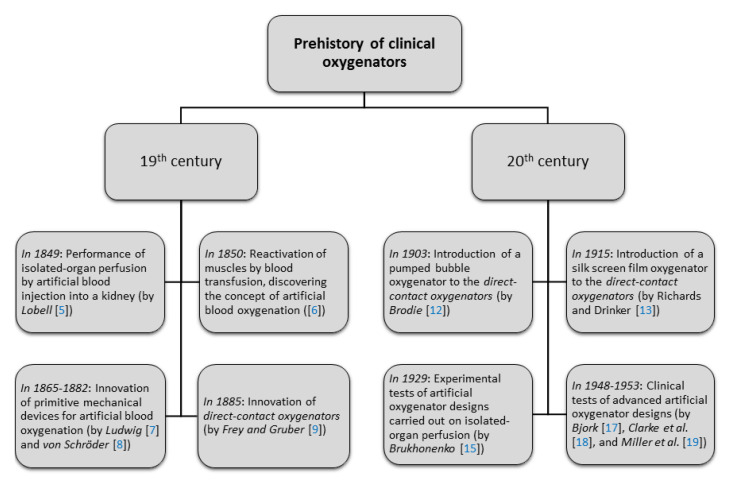
Historical milestones in the evolution of lung assist technologies.

Advancements to direct-contact oxygenators in the early twentieth century included a pumped bubble oxygenator by Brodie [12] and a silk screen film oxygenator by Richards and Drinker [13]. Experiments to test artificial oxygenator designs focused on isolated-organ perfusion, such as that by Hooker [14], who investigated the effects of pulse pressure on kidney function. By 1929, however, Brukhonenko [15] managed to successfully perfuse decapitated dog heads for several hours by passing blood through the lungs of a donor dog. Brukhonenko’s experiments demonstrated the potential for whole-body perfusion, but the use of animal lungs as oxygenators, being fragile and capable of transmitting infection, was abandoned in lieu of direct-contact oxygenators of increasing effectiveness [16]. In fact, by the mid-twentieth century, improved designs by Bjork [17], Clarke and Gollan et al. [18], and Miller and Gibbon et al. [19] were poised to enter the clinical environment. These historical development as depicted in Figure 2, marks the foundation of next generation lung assist technologies.

### 2.2. Direct-Contact Oxygenators for Clinical Usage

The first artificial oxygenator to see therapeutic use was that designed by American physician John H. Gibbon Jr [20]. Gibbon pioneered the development of a heart-lung machine: a device capable of oxygenating blood that had been detoured away from the heart and lungs [21]. In 1953, Gibbon’s heart-lung machine successfully excluded a human heart from normal circulation for 25 min, allowing the surgical repair of an atrial septal defect [22]. In Gibbon’s intricate device, blood was exposed to oxygen as it traveled down an array of stationary wire screens [10]. Modifications to Gibbon’s device by researchers at the Mayo Clinic in Rochester Minnesota resulted in the commercially-available Mayo–Gibbon pump-oxygenator [11,23]. Although operation of the device required a specially-trained surgical team, it was successfully used in conjunction with a number of intracardiac surgeries [24]. After a century of experimentation, an artificial oxygenator had for the first time been integrated into therapeutic medicine.

By the mid-1960s, two other direct-contact oxygenators had been standardized for clinical usage. The 1956 Kay-Cross disc oxygenator, developed by Kay and Cross et al. [25,26] was based off of Bjork’s [17] 1948 design. By using partially-immersed rotating disks, onto filmed venous blood, the design sought to increase oxygenation rates over stationary-screen methods. Despite having similar disadvantages to the Mayo–Gibbon pump oxygenator, which were being difficult to sterilize and requiring a large primer volume, the device received use in cardiac surgery for nearly two decades [27]. Receiving more widespread usage, however, was the 1955 DeWall bubble oxygenator. In DeWall’s design [28], derived from the bubble oxygenator described by Clarke et al. [18] venous blood was carried up a vertical column by a bubbly flow. Having been oxygenated, this foamy blood entered a chamber designed to coalesce the gas phase, and buoyancy forces separated the resulting bubbles from the blood as it continued down a helical tube. This design proved to be extremely efficient, and, as a disposable device, it required no re-sterilization [29]. By the 1970s, DeWall-type bubble oxygenators had become the artificial lung of choice, being used in an estimated 90% of open-heart operations worldwide [30,31].

Although a remarkable medical advancement, direct-contact oxygenators left much to be desired. The blood-gas interface was identified as a source of blood trauma, protein denaturation, coagulation disorders, and microembolisms [32,33,34,35], and the associated vascular problems ranged from deficient peripheral perfusion to progressive organ failure [36]. Such complications could typically be managed over the duration of intracardiac surgery [37], but for patients requiring longer-term lung pulmonary circumvention, direct-contact oxygenators were of little assistance. In general, total-body perfusion by means of a direct-contact oxygenator could only be safely sustained for 2–3 h [38]. Thus, while such oxygenators ushered in the era of open-heart surgery, further steps towards a semi-permanent artificial lung would come by different technologies.

### 2.3. Rise of Membrane Oxygenators

As direct-contact oxygenators were becoming standard clinical tools in the 1950s, a new design, the membrane oxygenator, was undergoing its first experimental trials. In 1944, Kolff [39] observed that venous blood became oxygenated simply by running its course through dialyzing tubes. This surprising result meant that oxygenators need not include a blood-air interface, but rather blood and air could be separated by a semi-permeable membrane through which gas transfer occurred. The advantages inherent to such a membrane oxygenator were clear. Firstly, direct blood-air contact, a major contributor to blood trauma, could be avoided. Furthermore, over bubble oxygenators, membrane oxygenators would not be liable to cause air embolism, and over film oxygenators, the blood would not be exposed to harmful screens or metal components [40].

Membrane oxygenation, however, was not without limitations. Membranes were found to inhibit oxygen transfer rates significantly, and early membrane oxygenator designs could not keep pace with the blood flow rates of human adults [41]. The first experimental membrane oxygenator, by Kolff et al. [42] in 1955, used polyethylene membranes, while ethylecellulose was used in the first clinical application of a membrane oxygenator in 1958 [43]. These relatively impermeable materials required that very large membrane surface areas (up to $25\;m^{2}$) be used in conjunction with large primer volumes. In the 1960s, polytetrafluoroethylene [44], followed by low-cost, highly-permeable silicone [45] became established as the membrane material of choice. In the same decade, the relationship between blood boundary layer distribution and gas diffusion was recognized, and considerable work went into designing new membrane geometries and active components for disturbing laminar flow. Benchmark designs include the capillary oxygenator by Bodell [46] which used hollow fiber membranes to control blood boundary layers, the flat sheet oxygenator by Bramson [47], and the deforming membrane oxygenator by Kolobow [48]. Many other designs were proposed, but into the early 1970s membrane oxygenators remained an experimental technology, with none attaining the required gas-transfer capacity and reliability needed for widespread clinical acceptance [49].

Motives to further improve membrane oxygenator designs were kindled when, in 1971, Hill [50] reported the first successful instance of prolonged extracorporeal life support by a membrane oxygenator, in which a man was perfused by a Bramson membrane lung for 75 h. To further increase gas transfer rates, microporous membranes were introduced [51,52], and new techniques for active mixing were proposed [53,54]. Physiological advances occurred as well; oxygenators could be connected to the heart via arteriovenous (AV), venovenous (VV), and venoarterial (VA) routes, but during the first clinical applications of membrane oxygenators, there was little agreement as to the best arrangement. In the 1970s, however, VA perfusion, allowing the most flexible hemodynamic support, was established as the preferred method [55]. Increased commercialization notarized the improving reliability of membrane oxygenators [56], and for the first time the reign of bubble oxygenators was challenged [57].

### 2.4. Towards Prolonged Life Support

By the end of the 1970s, two commercialized membrane oxygenators were utilized in approximately 18% of all cardiac surgeries in North America [58]. However, the ability of membrane oxygenators to sustain prolonged life support integrated the medical world the most [59,60], and the term extracorporeal membrane oxygenation (ECMO) was applied to such procedures. In 1975, the National Institutes of Health commissioned a multi-center clinical trial to test ECMO as a prolonged life support technique for patients with adult respiratory distress syndrome (ARDS). The conventional treatment, mechanical ventilation, was known to induce pulmonary barotrauma, and it was hoped that ECMO could circumvent such difficulties by allowing the lungs to rest. The report [61], however, was disheartening; ECMO treatments resulted in very high mortality rates. Subsequent research on extracorporeal life support in adults effectively ceased for a decade [38]. Further development of membrane oxygenators, however, was not abandoned. Interest in membrane oxygenator life support of neonates remained [62], a more effective replacement for bubble oxygenators was still desired [63], and the potential for ECMO to assist lung transplant patients had been identified [64].

In response to the continuing need for improved oxygenation techniques, membrane oxygenators underwent critical technological advances throughout the 1980s. Several refined classes of oxygenators resulted from improved membrane biomaterials and fluid mechanics understanding [65], Used for long-term respiratory support, the SciMed-Kolobow passed blood between the spiral windings of a gas-containing silicone envelope [66]. High flow resistance deformed the membrane envelope, and the resulting boundary irregularities enhanced gas transfer. For intervention in cardiopulmonary bypass, the Shiley M-2000 [67] used screen spacers to induce mixing inside tightly-packed layers of membrane-insulated flows [68]. Rather than sandwiching layers of gas and blood flows, other designs, such as the Bentley BOS series [69], returned to the capillary oxygenator technique of the 1970s, in which blood is passed through thin fibers exposed to gas cross flow. In these hollow fiber oxygenators, the use of microporous polypropylene successfully prevented thrombus formation [70], a problem that plagued silicone-based fibers previously explored [71]. Criss-crossing the fibers and internalizing the gas flow (rather than the blood flow) was found to induce effective, but gentle mixing of the blood [72]. Progress was also made in the design of active mixing oxygenators, an approach with diverse predecessors from the previous decade [73]. A successful active design by Bellhouse et al. [74,75] induced vortical blood flow to enhance mixing, and entered clinical markets as the Interpulse membrane oxygenator.

Overall, designs from the 1980s were more convenient, having integrated venous reservoirs and heat exchangers, and more cost-effective, having borrowed from hemodialyzer manufacturing methods. Furthermore, membrane oxygenator technology was revolutionized by microporous hollow fiber designs, which required only small priming volumes and allowed for highly efficient gas transfer [76]. By the 1990s, microporous hollow fiber oxygenators with internalized gas flow had completely uprooted the clinical presence of bubble oxygenators.

While such advances were readily applied to cardiopulmonary bypass procedures, providing prolonged extracorporeal life support remained a challenge for membrane oxygenators. Microporous membranes were unfortunately prone to plasma leakage after long-term usage [77]. This was caused by the formation of a hydrophilic layer on the membrane from phospholipid adsorption [78]. As a result, solid silicone oxygenators, being less efficient (and hence having larger priming volumes), were used for extracorporeal life support [79]. Clinical trials through the 1980s waded through these difficulties. In lieu of the original failure of ECMO to treat ARDS, Kobolow et al. [80,81] proposed that membrane oxygenators could be used in conjunction with mechanical ventilators: through a veno-venous partial bypass, the oxygenator would be solely responsible for carbon dioxide removal, while low-volume mechanical ventilation would be responsible for gentle oxygenation of the lungs. However, several clinical trials [82,83,84] of this technique, extracorporeal carbon dioxide removal (ECCO${}_{2}$R), failed to show that survival times improved over standard mechanical ventilation. For neonatal respiratory distress syndrome, the outlook was brighter. Clinical trials [85,86,87] demonstrated successful extracorporeal life support through ECMO for newborn infants. By 1987, the lung rest afforded by ECMO averaged an 81% survival rate for newborns with severe respiratory failure, and the treatment was in place in 18 neonatal centers across the United States [88].

For the adult population, however, ECMO treatments were met with only limited clinical success throughout the 1990s. Membrane oxygenators were sufficient for the operating room, but they were poorly equipped to keep up with adult gas exchange demands for long-term life support [89]. Furthermore, ECMO remained an extremely expensive and complicated procedure, requiring specially-trained clinicians to carefully monitor hemoglobin levels, platelet counts, and activated clotting times, and take various measures to avoid infection, hemorrhaging at cannulation sites, intracranial bleeding, and acute renal failure [90]. Such challenges of extracorporeal life support strongly reminisce those experienced in the early days of whole-body perfusion for intracardiac surgery.

### 2.5. From ECMO to Artificial Lungs

If ECMO was ever to become a safe and practical clinical procedure, it was clear that considerable changes would be needed. Indeed, since the 1990s, ECMO has undergone an array of improvements that have gradually redubbed it as an “artificial lung”. While the clinical ECMO during the 1980s almost exclusively used the Kolobow–SciMed lung [91], the approaches towards extracorporeal life support would see a greater diversification in both technology and methodology during the following decades.

Since the 1990s, three major advances in ECMO technology have allowed extracorporeal life support for more widespread clinical application. First, it was found that heparin-coated oxygenator circuits could greatly reduce undesirable blood activation [92,93,94]. Heparinization of membrane oxygenators was originally used for cardiopulmonary bypass operations, but later integrated into ECMO circuitry [95]. Second, gravity-dependent roller pumps, which greatly constrained patient motion, were replaced by centrifugal pumps that offered smaller priming volumes coupled with minimized risks of hemolysis [96]. The most critical recent advancement of ECMO, however, has been the discovery of the polymethylpentene (PMP) membranes. Through the 1990s, the most commonly used membrane material remained silicone, which, although lacking efficiency, did not have the leaking problems of microporous polypropylene membranes [79]. PMP membranes, however, combined longevity with gas-transfer effectiveness [97], and are the principal membrane material currently in use today.

Additionally important to the post-1990 design of artificial lungs has been computational fluid dynamics (CFD). Numerical simulations can predict the effectiveness of various oxygenator designs, avoiding the expense of prototype construction or experimental testing. Only modern computing power, however, has allowed the complex oxygenator flow problem to be approached. In its most general form, this problem involves solving the mass transfer equations for oxygen and carbon dioxide, coupled with the momentum and mass-conservation equations for a non-Newtonian blood flow through an intricate geometry. The first applications of CFD to artificial lung design included the investigation of blood flowpath geometry in a commercial oxygenator [98], the optimization of a hollow fiber oxygenator with external blood flow [99], and the identification of oxygenator regions liable to induce thrombotic deposition [100].

## 3. Modern Technologies

### 3.1. Ecmo Today

While a number of current artificial lung technologies today can accurately be described as “extracorporeal membrane oxygenators”, we herein reserve the term ‘ECMO’ for pumped, temporary extracorporeal life support systems. It should be noted that this is not a universal naming convention. In fact, modern ECMO systems are themselves been referred to as “artificial lungs”, as justified by their newfound reliability and reputability. ECMO is far safer and simpler than it has been in the past. It can now be managed by a single bedside nurse, support patients for up to several months, and has a smaller likelihood of succumbing to bleeding and other complications [101]. Today’s ECMO technology and clinical practice bear only a stark resemblance to 20th century ECMO, and the discouraging ECMO trials of the 1970s have lost considerable relevance [102].

Modern ECMO technologies are available for partial and total respiratory support. In both cases, venous blood in the right atrium is directed outside the body through a large cannula and then pumped through a membrane oxygenator. In venoarterial (VA) ECMO, the oxygenated blood is returned to the aorta, hence putting the oxygenator in parallel with the heart and lungs. In venovenous (VV) ECMO, the oxygenated blood returns to the right atrium, forming a series connection. On the extracorporeal side, hollow fiber PMP membrane oxygenators are generally used in conjunction with centrifugal pumps. Body temperature is often maintained through heat-exchangers [101].

Clinically, ECMO is now a viable life support option for respiratory failure and cardiac failure patients of all age groups [103]. Furthermore, ECMO has seen recent success in bridging lung failure patients to transplantation [104]. A double lumen cannula for VV access has even enabled ECMO patients to ambulate [105]. Currently, bioengineering research in ECMO is looking into the design and testing of long-term, wearable ECMO systems [106].

While ECMO is revolutionizing critical care, it lacks several properties inherent to the ideal artificial lung. Modern ECMO systems are not implantable, are dependent on pumps, greatly limit patient mobility, and cannot be taken outside the care of a hospital. Furthermore, they do not replace the metabolic function of the lungs, and lack the longevity needed to be used as a permanent lung replacement. The development and evaluation of ECMO methods is today a highly active field of research, but progress toward the ideal lung replacements or lung assist devices may come through several alternative technologies, which we herein investigate.

### 3.2. Intravascular Lung Assist

Intravascular lung assist devices (ILADs) are a class of intracorporeal, implantable membrane oxygenators for partial lung support. These devices supplement natural oxygenation and/or carbon dioxide removal, rather than totally circumvent lung function, and arrived out of an effort to reduce the complications of extracorporeal oxygenation. ILADs must be small enough to fit into intravascular regions, yet have enough surface area to allow sufficient gas transfer. Furthermore, as ILADs do not contain an external pump, they must avoid causing large pressure drops across the device [107].

The first intravascular oxygenator to be implanted in humans, IVOX, was introduced by Mortensen in 1989 [108]. This device, a bundle of hollow silicone membrane fibers, was inserted percutaneously into the vena cava. Analagous to extracorporeal hollow fiber oxygenators, oxygen was passed through the IVOX fibers and diffused into the surrounding blood flow. Due to size constraints, IVOX lacked the membrane surface area to provide sufficient oxygenation [109,110,111]. The static ILAD (S-ILAD), using a different fiber configuration, increased gas transfer rates over IVOX, but caused large pressure drops across the device [112].

To safely increase gas transfer rates, second generation ILADs turned to active components. Developed in the mid 1990s, the dynamic intravascular lung assist device, D-ILAD, rotated a twisted construction of hollow fiber membranes, inducing cross-flows and enhancing gas transfer [113]. Although effective at gas transfer, these rotating components were liable to cause damage to the vascular endothelium. In a less violent ILAD, the Hattler Catheter, a rapidly pulsating balloon placed within the membrane balloon enhanced gas transfer by generating radially-outward crossflows [114]. The Hattler Catheter improved on gas transfer efficiencies by 50% over the IVOX in animal studies, but its large size limited clinical relevance [115]. In 2009, further increases in efficiency were achieved through the use of impellers [116], but since then research on ILAD designs seems to have stalled. An ILAD having the proper size, gas exchange rates, pressure gradients, and biocompatibility necessary for clinical usage has not yet been developed.

### 3.3. Pumpless Extracorporeal Lung Assist

More successful at partial respiratory support have been pumpless extracorporeal lung assist (PECLA) devices, which reroute blood through a pumpless gas-exchanging device via an arterio-venous shunt. They can be connected between the femoral vein and the femoral artery in cases of hypercapnic respiratory failure [117], or between the pulmonary artery and the left atrium in cases of pulmonary arterial hypertension [118]. In recent practice, the Novalung device [119], a PMP hollow-fiber membrane oxygenator, is used for gas exchange. This device has seen increasing success at bridging patients to lung transplantation [120,121,122].

Modern PECLA is extremely effective at carbon dioxide removal, but it is only capable of oxygenating 15–20% of the total cardiac output [123]. Furthermore, PECLA increases cardiac workload and has the potential to induce limb ischema [124]. For these reasons, PECLA is not readily applied to patients with severe hypoxia or hemodynamic instability, in which cases VV ECMO is a more viable solution [104]. In the absence of these conditions, PECLA is a suitable alternative to ECMO in trauma patients with leading hypercapnic lung failure, but in general the use of VV-ECMO over PECLA is currently advocated [125].

The precise range of modern PECLA’s clinical applicability remains to be demonstrated through clinical trials. As a pumpless, carbon-dioxide removal device, however, PECLA represents an important advance in simplified respiratory assist technology, and may continue to see increased usage as a bridge to lung transplantation. To receive more widespread utilization, PECLA devices of the future must allow greater oxygenation rates and place less demands on the heart.

### 3.4. Total Artificial Lung

The total artificial lung (TAL) is intended to be a pumpless, single-unit version of the ECMO system for total respiratory support. The terms paracorporeal artificial lung (PAL) and thoracic artificial lung (also TAL) have also been used to describe such devices. These alternative terms more accurately describe the present state of TAL devices, which are not yet capable of completely replacing the natural lung. Thus far, TAL prototypes have been extracorporeal devices, but the long-term vision is towards an implantable device. Even in their extracorporeal form, however, TALs aim to place almost no limitations on patient mobility [126].

Analogous to the natural lung, blood entering the TAL is pumped solely by the right heart. Two modes of attachment have predominated TAL usage. For the in-series configuration, both the inflow and outflow are attached to the pulmonary artery, and the pulmonary artery is ligated between the two cannulae. For the in-parallel configuration, the inflow is attached to the pulmonary artery, while the outflow is attached to the left atrium. While the in-parallel configuration places less strain on the heart [127], blood is allowed to bypass the native lungs, which serve various metabolic functions and filter gas emboli [128]. The oxygenator, generally constructed from PMP or polypropylene membranes, is sometimes used in conjunction with a compliance chamber to better match natural lung impedance [129].

An enduring obstacle to successful TAL development has been the strict physiological constraints placed on the device. As it is powered by the heart, the device must closely match the impedance of the natural lung, lest right heart failure result. This proves challenging, for the device impedance is highly sensitive to adjustments in design [130]. Furthermore, the gas exchanger must be highly efficient to meet adult oxygenation needs, and must appropriately adapt to the patient’s state of physical activity. Finally, to be suitable for long-term usage, the TAL must invoke minimal blood trauma, and could ideally be operated without the use of anticoagulants.

Two TAL prototypes have recently been under experimental investigation. In the first, the BioLung (MC3, Inc, Ann Arbor, Michigan), blood enters the device through a central channel, and radiates outward through a bundle of hollow polypropylene fibers [131]. In 2007, the BioLung was found capable of maintaining 50–60% respiratory support for up to 30 days in ovine studies [132]. Further ovine studies in 2010 found that right ventricular dysfunction decreased cardiac output by 20–30% when 100% of cardiac output was directed through the device [133]. Aimed to reduce impedance, another model known as the compliant thoracic artificial lung (cTAL) accommodates varying stroke volumes through expanding compliance chambers on either side of a polypropylene hollow fiber oxygenator [134]. While maintaining sufficient gas exchange, cTAL has a lower flow resistance than previous TALs, but ovine studies recently indicated that, to avoid pulmonary hemodynamics, no more than 60% of cardiac output can be directed through the device [135].

Artificial lung thrombogenicity is among one of the key challenges for its prolonged use. Attempts are being made to coat the surface of the membrane with polymeric substances to reduce the clotting, coagulation, and plasma leaks [136,137,138,139]. Surface-generated nitric oxide (NO) is used to reduce the platelet activation and coagulation on fibers. Approach [140] using PDMS hollow fibers embedded with copper nanoparticles (Cu NP) infused into the NO donor S-nitroso-N-acetyl-penicillamine to reacts with Cu NP to generate NO has shown potential. Although a reduction in blood clotting is observed, it dampens the oxygen transfer by 13.3% compared to baseline. Poly-carboxybetaine coatings have also shown promising results in reducing protein and platelet-fouling. A reduction in fibrin formation and gross thrombus formation by 59% was effectively observed in animal studies [141]. Inhibition of coagulation factor XII (FXII) to knock-out thrombosis without causing abnormal bleeding is also found effective [142].

Computational modeling remains an important aspect of current TAL development. Advanced CFD enables the accounting for delicate physiological constraints of the TAL in ways not possible in previous decades. Recently, the effects of fiber arrangement on blood flow characteristics have been investigated [143], oscillating fiber bundles for increased gas transfer have been explored [144,145,146], and porous media models have been used to study device pressure drops [147].

Total artificial lungs are, perhaps by definition, the future of artificial lung technology. To attain the status of an implantable, pumpless, lung-replacement, considerable work remains to be done. In particular, future TAL designs must allow greatly increased gas transfer, be more compact, and have less impedance than their predecessors. It may take a combination of new biomaterials, design innovations, and rigorous computational testing, before such a device is ready for human trials.

### 3.5. Artificial Lung Microtechnology

Microtechnology may be integral to the next generation of artificial lungs. To reproduce the super effective gas-transfer mechanisms of the natural lung, microsystems that mimic an alveolar-capillary interface have been developed [148]. While capillary vessels are generally 5–10 μm in diameter, the characteristic width of blood passages in modern artificial lungs is 200–300 μm, meaning that transverse mixing is needed to bring red blood cells near enough to the gas exchange surfaces [149]. The small width of blood passages in microfluidic artificial lung technology precludes the need for transverse flows, hence blood trauma as well as the device size can be reduced.

There has been recent development of several preliminary microfluidic oxygenators [150]. Typically they are constructed from gas-permeable poly(dimethylsiloxane). Blood and oxygen travel through adjacent channels, which may be straight [151] or branching [152,153,154]. Some microfluidic oxygenators can take ambient air, rather than 100% oxygen, as an input [155,156]. While not presently ready for clinical applications, microfluidic oxygenators are a rapidly improving in gas exchange rates, compactness, and thrombogenicity. Detailed elemental modeling and physical insight into gas transport mechanisms as recently demonstrated by Ukita et al. [157], will pave the way for further development of microfluidic lung devices.These experimental devices have the potential to become the clinical oxygenator of choice, both for implantable and extracorporeal applications.

## 4. Portable Ventilators

The ARDS treatment is challenging and accounts for high mortality and morbidity rates [158]. Treatment modality of ARDS is delicate, as it requires to balance or restore the physiological needs of the body. A mechanical ventilator plays a vital role in the management and treatment of ARDS. Although these ventilators are successful in maintaining the basal gas levels in the body, they are known to produce reversible or non-reversible physiological conditions, detrimental to patients [159]. Current commercial ventilators used in hospitals are manual in terms of sensing patient condition, non-ambulatory, expensive, and rely on caregiver/clinician’s continuous involvement and experience for treating ARDS.

**Figure 3:**
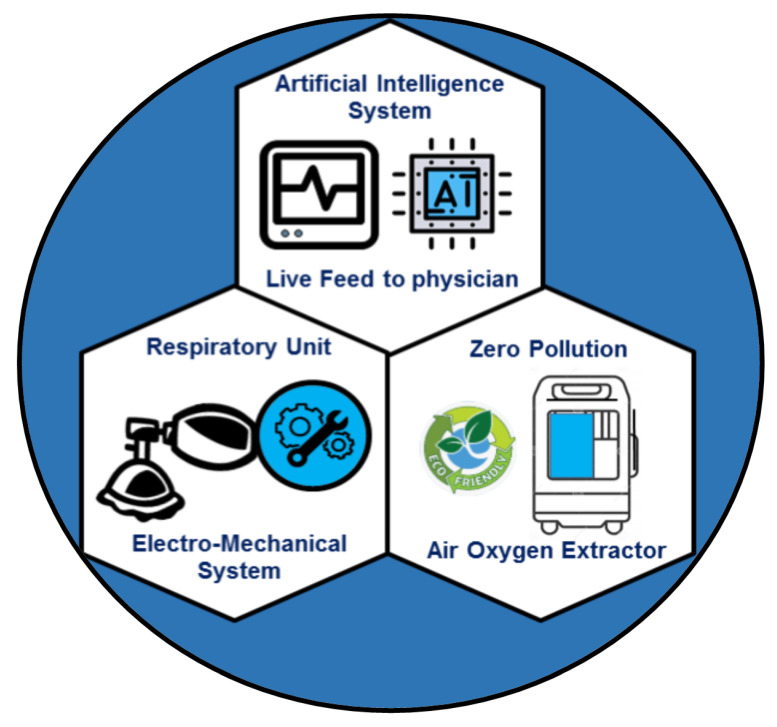
Schematic of the core components of the VENTIBAG.

Few attempts [160,161,162] have been made to minimize the cost and portability of the ventilators with limited success. Recently, a novel portable ventilator design has been proposed that eliminates the non-ambulatory bottle-neck of the current ventilators. It aims to provide a low-cost, smart, and a portable ventilator under the brand name VENTIBAG [163]. The core component schematics of the VENTIBAG is presented in Figure 3. It comprises of three major components: (a) respiratory unit, (b) oxygen extractor, and (c) artificial intelligent controller.

The respiratory unit consists of the electro-mechanical components that provide the main breathing action. VENTIBAG provides a novel approach to deliver oxygen on the go concept. It generates pure oxygen onsite to support critical patients suffering from lung diseases in pandemics or non-pandemic situations. In commercial ventilators, pure oxygen supply has to be provided through wall ports or oxygen cylinders; thus, immobilizing patients. Ambulatory support to patients is essential when the patient is not critically ill and is ideal in a pandemic situation where patients can be supported at home rather than overcrowding intensive care units. The artificial intelligent controller is the core brain of the VENTIBAG. To sense the state of the patient continuously, it deploys various sensors on the patients that continuously feed data to the AI controller. The pressure waveform (including any change in lung compliance and lung resistance), blood oxygen level, blood pH and CO_2_ levels are continuously fed to a neural network, which is trained against the normal human physiological condition, that determines the feedback setting of the electro-mechanical respiratory and FIO2 units. For better intervention, the neural network analyzes sensor data on an hourly basis. However, depending on the severity of the treatment, this window can be easily changed through a touch screen setting. The VENTIBAG design offers a greater mobility and minimal human intervention for continuous lung support and is deemed to be the future of ventilation and therapeutic lung support.

## 5. In Perspective

The 200-year history of artificial lung design is a remarkable tale of bioengineering innovation. The evolving terminology itself testifies to generations of progress: from the “perfusion apparatus”, to the “oxygenator”, to “extracorporeal life support”, to the “artificial lung”. While contemporary perfusion technology bears little resemblance to the rudimentary metallic devices of the nineteenth century, there exists a fascinating line of descendancy between them.

The modern challenges facing artificial lungs gas exchange rates and blood trauma have plagued such technologies since their inception. While extraordinary advances have been made, neither of these vices have been decisively and simultaneously overcome. However, now more than ever do artificial lungs aim to imitate the likeness of the natural lung, and this signals that viable lung replacement technology is near at hand. As bioengineering research labors towards that end, it is not to be forgotten that already has a great victory been won: perfusion technology is saving lives.
